# Comparison of mast cell counts between the patients with moderate and severe periodontitis

**DOI:** 10.15171/japid.2019.006

**Published:** 2019-08-31

**Authors:** Shirin Fattahi, Mehrnoosh Sadighi, Masoume Faramarzi, Elham Karimifard, Amirali Mirzaie

**Affiliations:** ^1^Department of Oral and Maxillofacial Pathology, Faculty of Dentistry, Tabriz University of Medical Sciences, Tabriz, Iran; ^2^Department of Periodontics, Faculty of Dentistry, Tabriz University of Medical Sciences, Tabriz, Iran; ^3^Private Practice, Tabriz, Iran; ^4^Student Research Committee, Faculty of Dentistry, Tabriz University of Medical Sciences, Tabriz, Iran

**Keywords:** Mast cell, moderate periodontitis, severe periodontitis, toluidine blue staining

## Abstract

**Background:**

The role of mast cells in periodontal tissue degradation has been established. These cells can be efficient in the etiology of periodontitis by participating in gingival homeostasis and releasing cytokines and enzymes, resulting in connective tissue matrix breakdown. Therefore, the aim of this study was to compare the mast cell counts between patients with moderate and severe periodontitis.

**Methods:**

This case‒control study was performed on 15 subjects with severe periodontitis and 15 subjects with moderate periodontitis, who needed periodontal surgical treatment. Incisional biopsies were provided during periodontal surgery. Afterward, the mean counts of mast cells were determined after toluidine blue staining of the samples. Finally, data were analyzed with SPSS.

**Results:**

The results of this study showed that mast cell counts in severe periodontitis cases were lower than those in moderate periodontitis. However, there were no statistically significant differences between the two groups (P=0.611). In addition, the mean mast cell counts in males and females did not show a statistically significant difference (P=0.231), although the count was higher in female subjects.

**Conclusion:**

Based on the results, no statistically significant differences were found in mast cell counts between subjects with severe periodontitis and those with moderate periodontitis.

## Introduction

Periodontitis is a chronic inflammatory disease associated with bacterial infection.^1^ The disease is characterized by destruction of the periodontal ligament and gingiva and subsequently, alveolar bone loss.^2^ Periodontal diseases exhibit various stages, beginning from gingivitis and progressing to initial and severe periodontal disease.^3^ Periodontal pathogens and their products cause pathological changes but cannot contribute to the widespread development of periodontal disease.^4^ It has been indicated that the reaction of the patient's immune system has an important role in the destruction of periodontal tissues.^5^ Numerous types of cells, including mast cells with their key role, are involved in the pathogenesis of periodontal disease. Mast cells (MCs) are mononuclear cells originating from CD34^+^ precursors in the bone marrow.^5^ They differentiate and mature in the peripheral tissues.^6^ MCs are normal residents of the lamina propria of human oral mucosa and gingiva.^7^ They produce many biologically active substances, which allow them to actively participate in immune and inflammatory processes linked with periodontal disease.^8^ In their cytoplasm, they contain approximately 80‒300 granules which display metachromasia with toluidine blue staining. In advanced periodontal disease, intraepithelial MCs apparently play a key role; however, their biological significance is yet to be fully understood.^9^ In human periodontal disease there is an increase in mast cell counts; these cells might participate either in the destructive events or in the defense mechanism of periodontal disease via secretion of cytokines.^8^ Based on the distribution of mast cells, it might be inferred that in the evolution of periodontal disease there are significant dynamic differences in migration and localization of these cells.^10^ Increased mast cell counts have been reported in the gingiva as compared to other healthy tissues.^11^ However, Gemmell et al^12^ evaluated chronic periodontitis lesions with healthy or gingivitis samples and reported lower mast cell counts in periodontitis lesions. Contrary to Carranza^13^ and Dummett,^14^ Günhan et al^15^ showed a significant increase in mast cell counts in infected tissues in comparison to healthy samples. Considering the controversies reported in the literature, the aim of the present study was to compare the mast cell counts between patients with moderate and severe periodontitis.

## Methods

### Sample Size and Sampling

As reported in similar studies, considering the mean mast cell counts in cases of aggressive periodontitis (8.47±1.42) and chronic periodontitis (11.4±2.249), and also by considering a=0.05 and 80% power, 12 samples were selected for each group. To improve the validity of the study, the sample size was increased by 20% and 15 samples were included in each group.^16^

### The Target Population

This case‒control study was conducted on 30 tissue samples from the patients referring to the Department of Periodontics, Faculty of Dentistry, Tabriz University of Medical Sciences; 15 samples were collected from the subjects with severe chronic periodontitis (patients with clinical attachment loss [CAL] of ≥5 mm with bleeding on probing [BOP]), and 15 samples were collected from subjects with moderate chronic periodontitis (patients with CAL of 3‒4 mm with BOP).

### Procedural Steps

The present study was carried out on patients referring to the Department of Periodontics, Tabriz Faculty of Dentistry. The subjects participated in this study voluntarily after the procedural steps were completely explained to them and after they signed informed consent forms. None of the subjects were deprived of routine treatment due to participating in this study. The subjects were included after diagnostic examinations and by considering inclusion and exclusion criteria.^16^

### Inclusion Criteria

1. Presence of at least 20 teeth in the mouth, with 8 teeth exhibiting CAL of ≥3‒5 mm and PD of ≥4 mm.
2. Patients with moderate and severe chronic periodontitis, needing periodontal surgery.
3. Presence of ≥3 mm of attached gingiva at the site of surgery.

### Exclusion Criteria

The exclusion criteria were as follows: smoking, a history of any systemic disease and any conditions affecting the periodontium (including inflammatory conditions like desquamative gingivitis), pregnancy or breastfeeding, hormonal disturbances (menopause, puberty, contraceptive drugs), history of antibiotic use or periodontal treatment in the last 6 months and patients with fixed orthodontic appliances.

### Study Groups

Disclosing tablets were used by a postgraduate dentistry student, under the supervision of a specialist to ensure proper oral hygiene before surgery. Patients with moderate and severe periodontitis were assessed by a periodontist.

Diagnosis of moderate chronic periodontitis was established by clinical information and histopathological findings in hematoxylin‒eosin-stained samples which were presented by WHO. Periodontology guidelines were noted in classifying different types of periodontitis.^17^ Incisional biopsy samples were taken sub-marginally (1 to 2 mm) from the surgery site and placed in 10% formalin; 5-µm incisions were prepared from the paraffin block samples. The slices were placed on slides, deparaffinized and dehydrated by 90% alcohol. Then the samples were stained with 1% toluidine blue for 30 minutes. Then the samples were inserted inside a 37°C incubator and washed with phosphate buffer for 1 minute, instantly dehydrated with 70% and 96% alcohol and observed under ×40 magnification in 4 HPF areas under an optical microscope and the mast cell counts were determined.

### Statistical Analysis

SPSS 20 was used for statistical analysis of data at P<0.05. Kolmogorov-Smirnov test was used to assess the normal distribution of data; since the data were distributed normally, parametric tests were used to analyze differences in the mean mast cell counts between the two study groups. Data were analyzed using descriptive statistics (mean ± SD). Independent-samples t-test was applied to compare mast cell counts between the two groups.

### Ethical Considerations

All the subjects signed informed consent forms to be included in the study. The protocol of the study was approved by the Ethics Committee of Tabriz Faculty of Dentistry under the code IRCT201502077128N5.

## Results

This cross-sectional study was based on 30 patients: 15 patients with moderate periodontitis and 15 patients with severe periodontitis. Descriptive statistics of the subjects are presented in Table 1 & Table 2.

**Table 1:** Mast cell counts in moderate and severe periodontitis

	**Mast cell counts** **Mean ± SD**	**Age** **Mean ± SD**	**Female**	**Male**
**Moderate periodontitis (n=15)**	16.63±3.15	47.46±8.01	9 (60%)	6 (40%)
**Severe periodontitis (n=15)**	15.22±1.87	48.33±8.63	8 (53.33%)	7 (46.67%)
**P-value**	0.611	0.796		0.561

**Table 2:** Comparison of mast cell counts between males and females

	**Mast cell counts** **(mean ± SD)**	**Number**	**P-value**
**Male**	15.89±1.24	13	0.231
**Female**	20.86±2.38	17	

The results showed that mast cell counts in the moderate periodontitis group were higher than those in the severe periodontitis group. The mean age of the subjects with moderate periodontitis and severe periodontitis in this study was 47 and 48 years, respectively. The number of female subjects with moderate periodontitis and severe periodontitis was 9 and 8 patients and the number of male subjects with moderate periodontitis and severe periodontitis were 6 and 7, respectively. Gender distribution between patients with moderate and severe periodontitis was not significantly different (chi-squared test, P=0.561).

**Figure 1:**
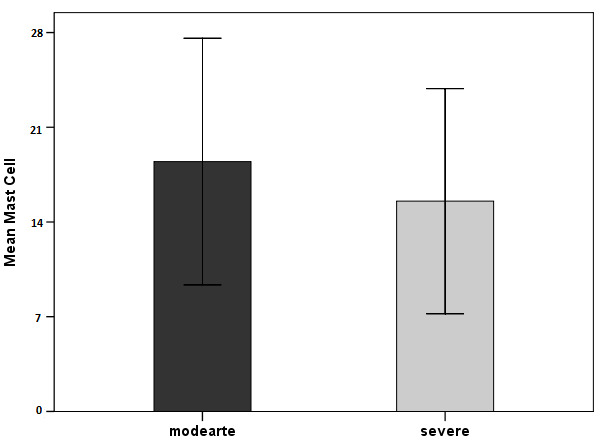
Comparison of mast cell counts between the two groups.

**Figure 2:**
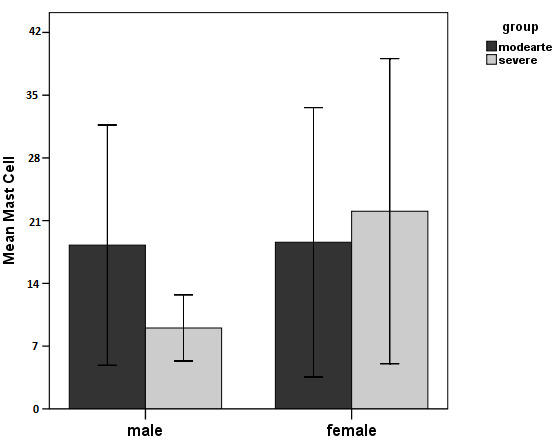
Comparison of mast cell counts in terms of gender.

Independent t-test showed no significant difference in the means of mast cell counts between moderate and severe periodontitis groups (P=0.611) (Figure 1 and 2).

## Discussion

Due to limited studies on the role of mast cells in periodontal diseases and the inconsistent results in previous studies, in this study we tried to investigate the effects of these cells on the severity of inflammation of periodontal tissues by examining a variety of periodontal diseases. This study is important since the results can justify diagnosis and management of periodontal patients and the association of this disease with inflammatory cells. In addition, the results can be used in the treatment of these patients.

Periodontitis is a multifactorial disease initiated by the microbial plaque, but its extent and severity depend on environmental factors, acquired diseases and genetic predisposition.^18^ It is one of the most common diseases of the oral cavity. Periodontitis is a periodontal tissue inflammatory response to dental plaque microorganisms that can lead to the degradation of gingival tissues and progression of the disease by increasing the depth of the periodontal pocket and reducing the supporting tissues of the tooth, bleeding, abscesses and toothache, ultimately leading to tooth loss.^19^

Studies on mast cells in different normal and pathological conditions have shown that mast cells are complex, well-organized and multi-functional cells that play a key role in innate and acquired immunity.^20^ Among cells found in periodontal diseases, mast cells are found in varying amounts in both inflammatory and healthy areas.^13^ The response of mast cells to periodontitis has been studied in several studies and conflicting results have been obtained. Some researchers have reported a decrease in the number of these cells, although some have seen significant increases in these cells.^21,22^ The relationship between the number of mast cells and periodontal health has been reported as increasing or decreasing number of mast cells in the inflamed tissue. The inhibition of mast cell release has led to a reduction in bone loss and a reduction in mast cell counts in tissues with chronic periodontitis as compared to gingivitis.^23^

Romana et al^18^ examined the role of mast cells in periodontitis and showed that mast cell counts in periodontal disease increased significantly compared to the healthy condition, with the difference that the density of mast cells in moderate periodontitis was lower in comparison with severe periodontitis. The difference can be attributed to the different roles of mast cells.^18^ In our study, although mast cells were more numerous in patients with moderate periodontitis patients compared to those with severe periodontitis, the difference was not statistically significant.

Lagdive et al^8^ investigated the relationship between the mast cell counts and periodontal diseases and reported higher counts of mast cells in chronic periodontitis and gingivitis. They also reported a significant difference in the gingivitis and chronic periodontitis in mast cell counts. Furthermore, in the periodontitis group, there was a significant increase in mast cell counts, which indicated the significant role of mast cells in tissue degradation in chronic periodontitis.

Batista et al^21^ evaluated mast cell counts at different stages of periodontitis. The results of immunohistochemistry staining showed higher mast cell counts in chronic periodontitis and gingivitis in comparison with healthy gingival tissue. The researchers pointed out that mast cell counts in periodontitis could increase through participation in tissue degradation or in the defense mechanism of periodontitis, through the secretion of cytokines, Th2 responses, cell migration and healing processes.

Vahabi et al^24^ investigated the relationship between the mast cell counts and chronic periodontitis. The results of the study showed that the mast cell counts in the group with chronic periodontitis were higher than the group with invasive periodontitis and healthy group. Individuals with acute periodontitis did not exhibit higher mast cell counts than healthy subjects. In that study, there was no correlation between the degree of inflammation and mast cell counts, consistent with the results of the present study. In the present study, although the mast cell counts in the severe periodontitis group were low, the difference between the two groups was not statistically significant. In the study above, they proposed more extensive studies with more precise methods to clarify the role of these cells in the pathogenesis of chronic periodontitis. It was also suggested that the aspects of host dynamic defense be investigated along with other aspects of the immunity system.

The reason for the inconsistent results concerning mast cell counts in moderate and severe periodontitis might be the different methods of counting cells in different studies. Another possible explanation for the higher number of mast cells in moderate to severe periodontitis can be related to the multiple roles of these cells. Mast cells in periodontal diseases not only play the role of inflammatory cells, but they also contribute to the angiogenesis. As a result, an increase in their number can be a primary phenomenon in the progression of inflammatory changes in the gingival tissue.

## Conclusion

the results of this study did not show a significant difference in mast cell counts between severe and moderate periodontitis samples. although the number of mast cells in females was higher, the mean count of mast cells was not significantly different between males and females. because of the importance of periodontal disease and the possible role of mast cells in different stages of periodontal disease, further studies are necessary to identify the cellular and immunological interactions involved in the disease so that their results can be used to apply effective therapy.

## Authors’ Contributions

The study was planned by SHF and MS. Data collection was carried out by EKF; statistical analyses and interpretation of data were carried out by SHF and EKF. The manuscript was prepared by AAM and MS and revised by MS. All the authors have read and approved the final manuscript for submission.

## Competing Interests

The authors declare that they have no competing interests with regards to authorship and/or publication of this paper.

## Ethics Approval

The study protocol was approved by the Ethics Committee in Medical Research, Tabriz University of Medical Sciences.
